# Designing Effective eHealth Interventions for Underserved Groups: Five Lessons From a Decade of eHealth Intervention Design and Deployment

**DOI:** 10.2196/25419

**Published:** 2022-01-07

**Authors:** Edmund WJ Lee, Rachel F McCloud, Kasisomayajula Viswanath

**Affiliations:** 1 Wee Kim Wee School of Communication and Information Nanyang Technological University Singapore Singapore; 2 Dana-Farber Cancer Institute Boston, MA United States; 3 Harvard T H Chan School of Public Health Boston, MA United States

**Keywords:** eHealth, mobile health, communication inequalities, health disparities, health informatics, mobile phone

## Abstract

Despite the proliferation of eHealth interventions, such as web portals, for health information dissemination or the use of mobile apps and wearables for health monitoring, research has shown that underserved groups do not benefit proportionately from these eHealth interventions. This is largely because of usability issues and the lack of attention to the broader structural, physical, and psychosocial barriers to technology adoption and use. The objective of this paper is to draw lessons from a decade of experience in designing different user-centered eHealth interventions (eg, web portals and health apps) to inform future work in leveraging technology to address health disparities. We draw these lessons from a series of interventions from the work we have done over 15 years in the Viswanath laboratory at the Dana-Farber Cancer Institute and Harvard TH Chan School of Public Health, focusing on three projects that used web portals and health apps targeted toward underserved groups. The projects were the following: *Click to Connect*, which was a community-based eHealth intervention that aimed to improve internet skills and health literacy among underserved groups by providing home access to high-speed internet, computer, and internet training classes, as well as a dedicated health web portal with ongoing technical support; *PLANET MassCONECT*, which was a knowledge translation project that built capacity among community-based organizations in Boston, Lawrence, and Worcester in Massachusetts to adopt evidence-based health promotion programs; and *Smartphone App for Public Health*, which was a mobile health research that facilitated both participatory (eg, surveys) and passive data (eg, geolocations and web-browsing behaviors) collection for the purpose of understanding tobacco message exposure in individuals’ built environment. Through our work, we distilled five key principles for researchers aiming to design eHealth interventions for underserved groups. They are as follows: develop a strategic road map to address communication inequalities (ie, a concrete action plan to identify the barriers faced by underserved groups and customize specific solutions to each of them), engage multiple stakeholders from the beginning for the long haul, design with usability—readability and navigability—in mind, build privacy safeguards into eHealth interventions and communicate privacy–utility tradeoffs in simplicity, and strive for an optimal balance between open science aspirations and protection of underserved groups.

## Background

Breakthroughs in communication technologies in the past decade have brought about a plethora of opportunities for researchers to leverage different forms of digital technologies to bridge health disparities. With the ubiquitous use of digital tools such as computers, laptops, and smartphones and recent advances in cyberinfrastructure to store and analyze big data at scale with efficiency, researchers have piloted and designed web portals to specifically aid health-information seeking among different populations [1,2], encouraged the use of patient portals for health care management [3], and developed smartphone apps and wearables to improve health monitoring [4].

## eHealth Interventions in Widening Inequality

Despite the benefits brought about by the communications revolution, a major critique in eHealth intervention research is that instead of closing the gaps between the rich and the poor, the introduction of digital gadgets reproduces and reinforces existing social structures of health inequalities [5-8]. For instance, research has shown that in the United States, compared with White and non-Hispanic individuals, Black and Hispanic individuals were significantly less likely to be offered access to web-based personal health information portals by their health care providers, and they were also less likely to use personal health information, even when granted access [9]. Although the combination of artificial intelligence algorithms and the availability of electronic health records (EHRs) promises to enable clinicians to predict hospital readmissions and mortality [10], people from lower socioeconomic groups may not necessarily benefit from these if their health records are not recorded in the EHRs in the first place because of multiple missed appointments [11]. In some instances, the places where people from lower socioeconomic groups seek treatment may not be equipped with EHRs. Even when people from lower socioeconomic groups have access to digital health technologies, they may not be able to afford the recurring expenses needed to use the services or maintain stable internet connections [12]. Even if access to these technologies is not an issue, underserved groups may find it difficult to navigate the complex platforms of different digital health technologies [13].

Beyond financial and technology factors, research has shown that another significant challenge to eHealth interventions is the lack of collective use and adoption by people’s social networks [6]. A clear example is in the current COVID-19 pandemic, where a major challenge in implementing and leveraging data from contact tracing apps is the low uptake by the general population [14]. The problem is compounded when people from marginalized communities and people of color, who have been hit the hardest by the pandemic, remain skeptical of technology because of the historical baggage of being unfairly targeted by state surveillance or being wary of the amount of misinformation and disinformation generated from these digital sources [15,16].

In today’s world, where data collected from eHealth interventions can be used to understand health behaviors when individuals from underserved groups do not use the digital gadgets designed for them and are systematically left out, it contributes to a larger problem called *data absenteeism*—missing data of underserved groups in health systems—leading to inaccuracies and perpetuation of biases when training machine learning models on these data sets with large amounts of missing data [17].

## The Need to Address Inequality in eHealth Interventions

To level the playing field in ensuring that eHealth interventions benefit underserved groups, it is paramount that researchers and public health organizations pay attention to the underlying mechanisms that contribute to *communication inequalities*. Communication inequalities are structural, interpersonal, and individual differences among social groups in accessing, using, and processing information from media and communication technologies, and they mediate the relationship between social determinants and health outcomes [18]. Thus, although investing in the usability of eHealth interventions is important, it is crucial that public health scholars go beyond technological considerations and examine how external, social, and individual contexts synergistically and collectively influence the success of eHealth interventions [17]. After all, decades of public health research have fervently called for the need to engage individuals, social networks, the broader community, and anchor institutions (eg, hospitals and community health care organizations) in implementing health promotion programs [19,20]. The purpose of this paper is to showcase and draw lessons from over a decade of our research experience in implementing different eHealth interventions among underserved groups.

## A Framework for Addressing Communication Inequalities: Structural Influence Model

**Figure 1:**
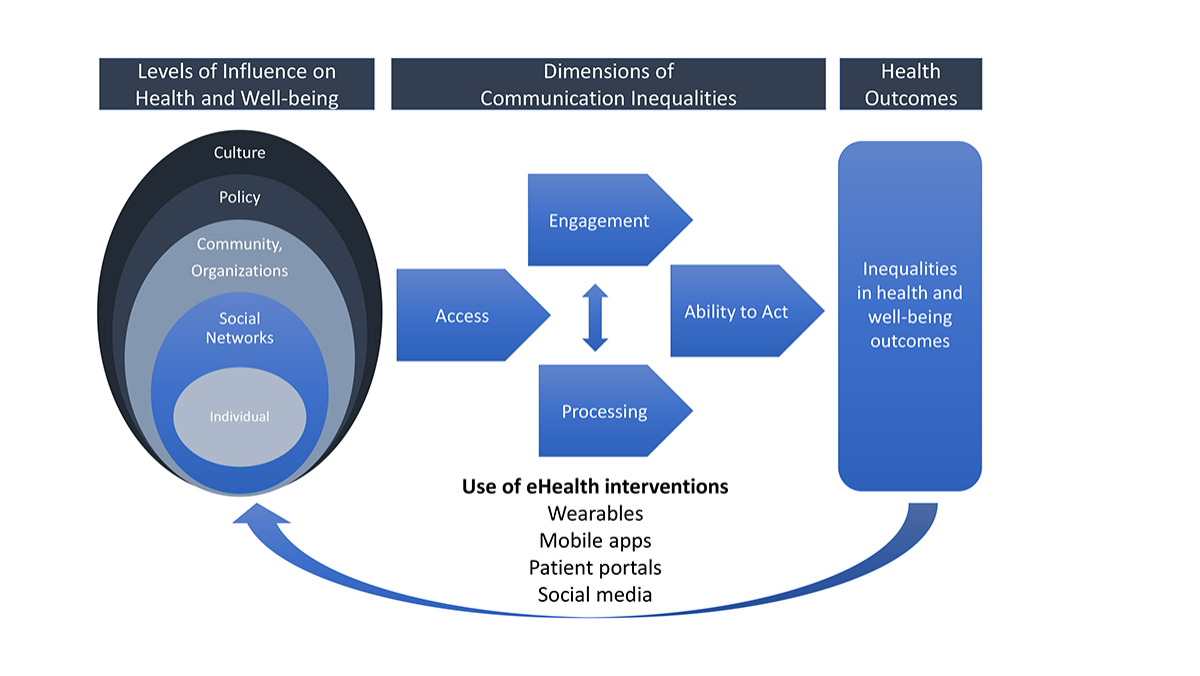
Modified structural influence model.

Our work has focused on examining information and technologies that can help bridge unequal health outcomes between different social groups, guided primarily by the structural influence model of communication (Figure 1), which delineates the relationship between how communication inequalities mediate and moderate the association between social determinants and communication and health outcomes [18,21]. The framework postulates that the use of eHealth interventions can have both direct and indirect impacts on health, and they may potentially amplify disparities among different social, racial, or ethnic groups. Central to the framework is the concept of communication inequalities, which is defined as unequal access and use of communication technologies because of differences in how people access, use, and process information from digital media platforms, which widens existing health disparities [7,17,22]. In other words, the fundamental assumption is that whoever has the power as well as the cognitive, social, and financial resources (typically, people with higher income and education) to access, use, and process information from communication technologies is most likely to benefit, whereas groups that are underserved, even if they have access to such platforms, seldom reap the promised benefits though they are often the target audience of such interventions [23].

Despite the stark reality of health disparities, the silver lining is that communication inequalities are ultimately *modifiable* conditions that could be addressed by academic institutions, public health organizations, or government agencies more readily as compared with social determinants. To be effective, the design of eHealth interventions should consider broader contexts that may attenuate the uptake and use of communication technologies.

At the individual level, research has shown that underserved groups face significantly more barriers— economically, socially, and technologically—in the adoption of eHealth interventions when compared with their financially better off counterparts [24]. A study on the adoption of web-based patient portals found that although participants were given the opportunity to register for web-based patient portals, only 22% of patients with limited health literacy registered for the account. This was lower when compared with 73% of adults with adequate health literacy skills [25]. This pattern was consistent with racial differences as well, with minority groups being at higher odds of not using these eHealth interventions [26]. Beyond individual factors, external factors, such as individuals’ social networks, the type of hospitals in the community, and community-based organizations (CBOs) play a crucial role in boosting health literacy [27-32]. Thus, it will be strategic to gain insights into CBOs and social networks of the underserved groups, as they would have the experience in identifying the type of interventions that would best suit the needs of the underserved populations and those that would simply not work [33,34]. Without the input of the community, designers of eHealth interventions may fall into the trap of having *solutions in search of a problem* [35] and forcing innovations that simply do not work in the community.

The rest of this paper will illustrate the key lessons learned from three signature projects in the Viswanath laboratory. The first project was called Click to Connect (C2C), which was a community-based eHealth intervention that aimed to improve internet skills and health literacy among underserved groups by providing home access to high-speed internet, computer, and internet training classes, as well as a dedicated health web portal with ongoing technical support. The second project was PLANET MassCONECT, which was a knowledge translation project that built capacity among CBOs in Boston, Lawrence, and Worcester in Massachusetts to adopt evidence-based health promotion programs. Finally, the third project was Project Smartphone App for Public Health (SNAP), which was a mobile health research that collected both participatory (eg, surveys) and passive data (eg, geolocations and web-browsing behaviors) for the purpose of understanding tobacco message exposure in an individual’s built environment.

We have distilled five key principles on eHealth intervention research for underserved groups from these three projects: (1) develop a strategic road map to address communication inequalities (ie, a concrete action plan to identify the barriers faced by underserved groups and customizing specific solutions to each of them), (2) engage multiple stakeholders from the beginning for the long haul, (3) design with usability—readability and navigability—in mind, (4) build privacy safeguards into eHealth interventions and communicate privacy–utility tradeoffs in simplicity, and (5) strive for an optimal balance between open science aspirations and protection of underserved groups. We have also proposed multilevel strategies for developing eHealth interventions that health organizations could draw on in their work with underserved groups.

## Project 1: C2C

**Figure 2:**
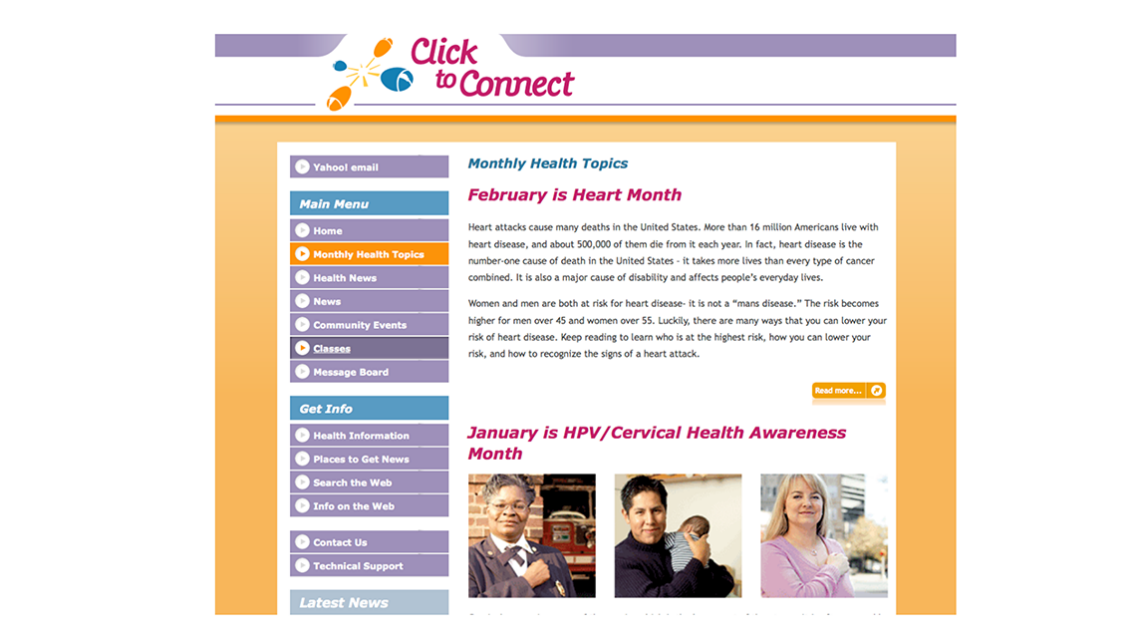
Screenshot of the web-based health portal of Click to Connect.

Project C2C was a randomized controlled trial that aimed to improve eHealth literacy among people from lower socioeconomic positions (SEPs). To achieve the objective of empowering people from lower SEP groups by taking advantage of web-based health portals to seek information and gain health knowledge, we designed an intervention that involved the (1) development of a web-based health portal (Figure 2) from scratch that was customized for novice or less experienced users to easily navigate and access the internet, specifically health information; (2) purchase and provision of computer and broadband internet access for the entire length of study; (3) training classes where participants were taught digital skills such as how to use computers and the internet; and (4) ongoing technical support if participants had any questions on the health web portal or connectivity issues [34]. The trial was conducted in three waves from 2007 to 2009, and for each wave, participants attended 9 monthly training classes at community colleges located in Boston. Participants randomized to the control group received health information at the end of the study period. Participants were recruited from adult education centers located in the Greater Boston area of the state of Massachusetts [36].

Researchers generally make insufficient efforts to reach underserved populations with the stereotype that they are *hard to reach*. We adopted a *proactive* approach, which involved arranging face-to-face contact with community leaders and organizations, as well as recruitment presentations and meetings in the community [37]. Overall, we made a total of 190 in-person presentations at 32 adult literacy centers in Greater Boston from May 2007 to October 2009.

## Project 2: PLANET MassCONECT

Although project C2C was targeted at individuals from underserved groups, project PLANET MassCONECT was a 5-year knowledge translation project funded by the US National Cancer Institute that aimed to build the capacity to find, adapt, and implement evidence-based health promotion programs among a diverse group of CBOs located in three cities in the state of Massachusetts (Boston, Lawrence, and Worcester) in the United States [38]. In other words, PLANET MassCONECT aimed to strengthen CBOs by creating a network where organizations could collaborate with other CBOs, researchers, and community members and provide training and technical support to use a customized web-based health portal to find relevant health resources and data to implement evidence-based health promotion projects. At the end of the training, CBOs were plugged into a network with other CBOs and encouraged to apply for mini-grants to strengthen interorganizational collaboration in addressing health disparities [39].

These aims were achieved through an intervention that included these main components: (1) development of a web-based health portal containing health resources, including evidence-based interventions, data, and resources for interventions; (2) a 2-day capacity-building workshop for CBOs; (3) provision of training manual, handouts, and case studies; (4) highlighting pilot grants to apply newfound knowledge; and (5) facilitating networking opportunities to promote learning networks in which trainees can support each other.

**Figure 3:**
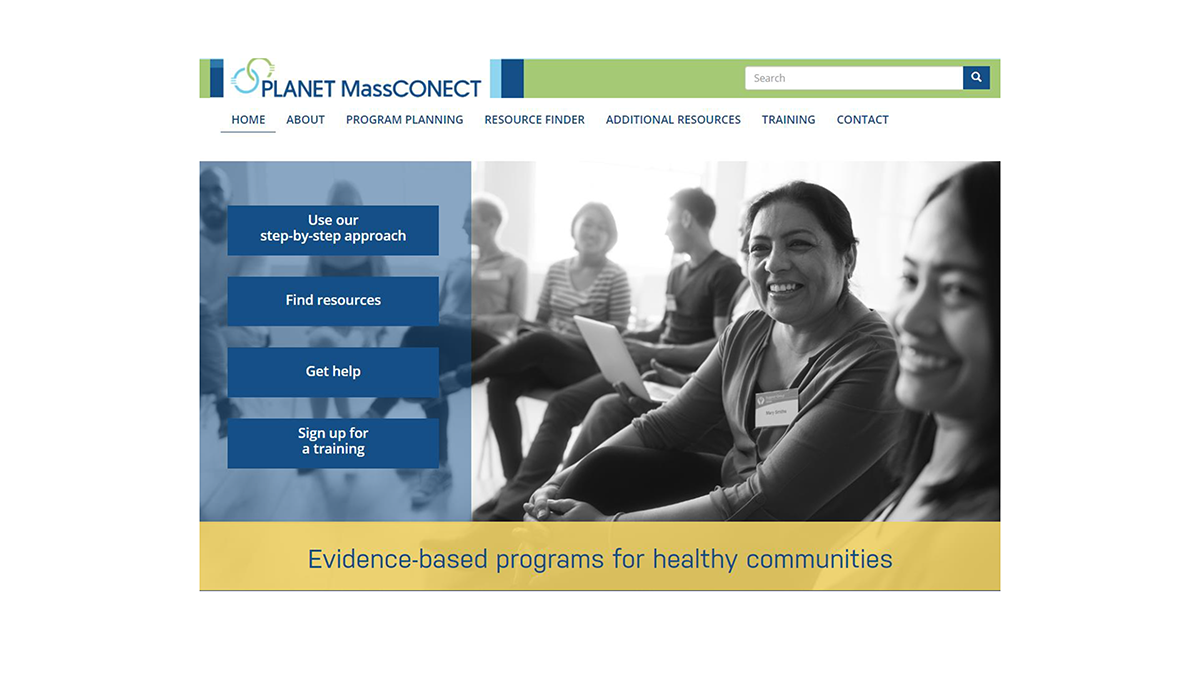
Screenshot of the web health portal of PLANET MassCONECT.

The web-based health portal (Figure 3) was meant to serve as a *one-stop dissemination marketplace*, which would contain localized health resources to aid health program planners in CBOs to systematically implement evidence-based interventions in their communities. Participants in our project were staff of CBOs in the 3 cities if they met the following criteria [39]: (1) aged ≥18 years, (2) working for CBOs (ie, nonprofit or public service sector organization) in one of the three cities (Boston, Lawrence, and Worcester), and (3) actively involved in developing health promotion programs and efforts in their CBOs.

## Project 3: SNAP

Project SNAP was a smartphone-based study that was a collaborative effort between the Dana–Farber Cancer Institute, the University of Saskatchewan, and Baylor College of Medicine and was funded by the Truth Initiative. The objective of the study was to leverage smartphone capabilities in collecting data both passively (eg, collecting geolocation, web browsing, and Bluetooth data) and actively (eg, prompting respondents to answer surveys) to measure exposure to tobacco messages in a real-world setting among participants from underserved groups. Our collaborators from the University of Saskatchewan developed a smartphone app called *Ethica*, which captured complex, rich message exposure data that could use multiple sources to provide information above and beyond what is available in traditional surveys. The app captured data such as (1) responses to ecological momentary assessments in the form of short surveys containing questions on health-information seeking, quit attempts, message exposures, and interpersonal communication regarding tobacco products; (2) photos of pro- or antitobacco messages in the environment; (3) tracking of their web-browsing behaviors; and (4) geolocations.

These three projects were chosen (out of many others in the laboratory) because they each focused on how different forms of eHealth gadgets could close gaps in health disparities. For instance, C2C and PLANET MassCONECT examined the efficacy of using web-based health portals to facilitate health information seeking, whereas SNAP explored the effectiveness of using smartphone apps to capture in situ data (eg, surveys, geolocations, and web search browsing) to understand how external and information environment influence tobacco consumption. Also, these three projects were selected because of the diversity of the categories of participants. For instance, the web-based health portal for C2C was targeted at individuals from underserved groups, whereas the site for PLANET MassCONECT was designed for CBOs that work with underserved groups to find and extract data to promote evidence-based health promotion. SNAP, unlike C2C and PLANET MassCONECT, used smartphone-based methods to examine environmental exposure to tobacco messages among youths of low SEP.

## The Five Principles for eHealth Interventions

Our experience in working with underserved groups in these digital health projects allows us to draw lessons for effective eHealth intervention research. These lessons were derived primarily from a series of focus groups and usability tests of the eHealth interventions that we conducted for each of the projects, as well as from feedback from community partners. Here, we condensed and extracted five principles: (1) develop a strategic road map to address communication inequalities, (2) engage multiple stakeholders from the beginning for the long haul, (3) design with usability in mind,—enhancing readability and navigability—(4) build privacy safeguards into eHealth interventions and communicate privacy–utility tradeoffs that come with simplicity, and (5) strive for an optimal balance between open science aspirations and protection of underserved groups. Multimedia Appendix 1 summarizes the objectives of each project, the target groups, and the lessons and principles learned.

## Principle 1: Develop a Strategic Road Map to Address Communication Inequalities

**Figure 4:**
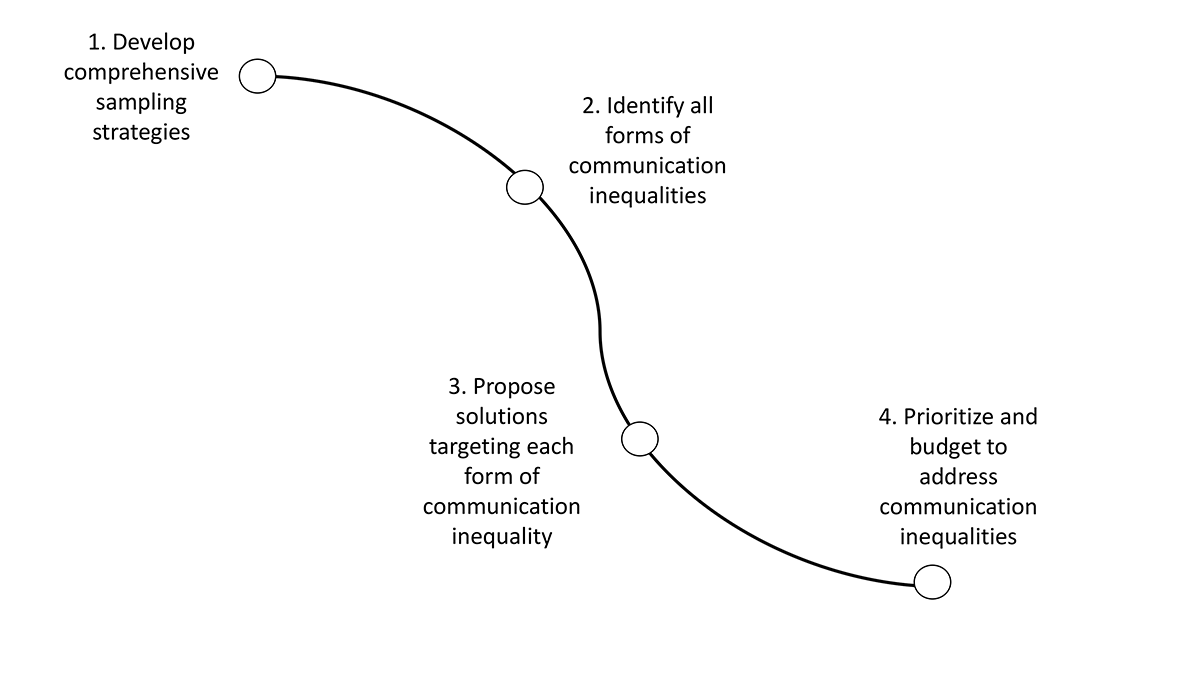
Strategic road map for addressing communication inequalities.

First, one of the most important lessons is that it is crucial for research teams to intentionally put in place a strategic road map detailing specific approaches to reduce the burden posed by different forms of *communication inequalities* even before designing eHealth interventions. The presence of communication inequalities poses significant barriers to the underserved population regarding taking advantage of health benefits from using eHealth interventions [40]. Decades of research have documented that even when presented with the same technologies or health information, the ones that benefited the most were people from higher SEPs, whereas people from lower socioeconomic groups were consistently left behind. It goes to show that no matter how sophisticated or cutting edge the technologies are, eHealth interventions are not magic bullets. Instead, introducing eHealth interventions without understanding the broader context of underserved groups will exacerbate the inequalities and preclude people from lower socioeconomic groups from taking advantage of health technologies. As such, without paying attention to what these communication inequalities are, implementing eHealth interventions may inadvertently widen gaps in health inequalities. Although the idea of a strategic roadmap in itself is not novel, there are very few that have explicitly championed the need for a roadmap when working with the underserved [7]. Therefore, what should go into the strategic roadmap? We highlight four key components (Figure 4) to (1) develop comprehensive sampling strategies; (2) identify all forms of communication inequalities, including contextual conditions, and understand the digital media landscape of underserved populations; (3) propose solutions targeting each form of communication inequality; and (4) prioritize and budget for alleviation measures.

First, researchers have to be cognizant of the fact that underserved groups may be either reluctant to participate or face barriers to participation in research studies. For instance, those from racial minorities may be wary of participation in experiments, given the poor experience and exploitation in the past, such as in the infamous Tuskegee experiment in which researchers withheld penicillin for the treatment of their participants’ syphilis, although the treatment was widely available [41]. Alternatively, day-to-day experiences with discrimination and poor treatment from those in power may color their experience regarding research. Several reasons have been given for this, such as involved participation risks (eg, participants may not want to identify with researchers’ labeling of fear of harm) and opportunity costs (eg, participating in research might mean less time for work or attending doctors’ appointment) [42]. To address these concerns, researchers should schedule in-person face-to-face contact with community leaders and organizations, as well as recruitment presentations and meetings in the community where they could present the objectives of the study, articulate how participation in research would benefit the community directly, and address queries and for the community to know the researchers in person.

Second, the road map should identify different forms of communication inequalities. Perhaps the most visible form of communication inequality would be the *lack of access* to these technologies, be it desktops, laptops, smartphones, or wearables [43], in addition to other factors, such as literacy level and the degree of confidence and efficacy in navigating digital media. Requiring participants from underserved groups to take a portion of their paycheck to purchase communication gadgets is nearly unthinkable, considering their financial constraints. Although this is intuitive for the most part and for many researchers working with urban or rural people from lower socioeconomic groups, what is less apparent is factoring in the hidden costs that underserved populations need to pay to maintain the connection and use of the device. This is known as *connection maintenance cost*, or recurring expenditure, which could refer to the additional financial, time, and energy resources that people from lower socioeconomic groups need to pay to use eHealth interventions [12,17]. For instance, people from underserved groups are more likely to face difficulties in dealing with technical difficulties and experience intermittent internet connection [44].

After identifying these factors, researchers need to propose tangible solutions to address each of these communication inequalities and reflect their commitment through their research budgets. For C2C and SNAP, we provided not only the actual devices required for interventions (eg, computers, web portals, and smartphone apps) but also paid for broadband internet access for participants to ensure that they do not face intermittent connectivity problems, which would dilute the effectiveness of the interventions. We saw some degree of success, especially for our C2C project, where we found that when provided with internet access, participants from underserved groups increased the use of the internet for capital-enhancing purposes, such as seeking health information, contrary to critics who assumed that underserved groups would only use the internet for entertainment purposes [45].

In addition, beyond identifying *deficits* in access to communication technologies, research teams and researchers could creatively design eHealth interventions by leveraging the communication gadgets that the underserved groups are already using instead of introducing or imposing newer forms of technologies that might be met with skepticism. For instance, instead of asking participants to purchase wearables for health tracking, which would be out of their budget, research teams could design health apps that achieve the same functions but at little to no cost to participants. For this reason, for our SNAP project, the Ethica app was repurposed to allow our participants to engage in participatory research on tracking exposure to tobacco messages in their external and web-based environment at no cost.

## Principle 2: Engage Multiple Stakeholders From the Beginning for the Long Haul

In the pursuit of developing technologies for underserved populations, the temptation is to develop a technological *messiah complex*, believing that the success and failure of eHealth interventions in improving health rest solely on researchers or the technology. Our experience in working with underserved populations through the years has demonstrated that even well-resourced institutions can be rendered ineffective if they adopt a lone wolf approach in the implementation of eHealth interventions among people from lower socioeconomic groups. Even large anchor institutions such as hospitals need to partner with both public and private organizations if they want to make a sustainable change in the community in which they are located [46], as they may not have a comprehensive understanding of the challenges faced by people in their neighborhood. As such, we advocate researchers to consider a participatory approach and involve and engage different stakeholders from the get-go when designing technology.

We found that engaging multiple stakeholders from the start is nonnegotiable in the development and implementation of eHealth interventions. Although researchers may possess the technological expertise (eg, developing web portals and programming apps for mobile phone solutions) and rigorous training in social-scientific theories, they do not have expertise on the contextual conditions in which the interventions are implemented. Such expertise lies with the users and stakeholders in the community. Depending on the context, there may be a lack of trust in the researchers (ie, underserved populations may have fears about how their data may be misused), uncertainty in how the results would be used (ie, profiling of racial minorities), or a lack of awareness and education regarding the benefits of incorporating the interventions [47]. Therefore, by identifying key stakeholders and forging long-term synergistic collaborations, researchers could benefit from stakeholders’ connections within underserved groups and provide scientific expertise that would be relevant in equipping and empowering stakeholders.

So, who are the key stakeholders, and what would be the best way to engage them? There are many types of organizations and individuals that can directly or indirectly influence the success or failure of eHealth interventions [20,48]. Here, we highlight three distinct groups: (1) CBOs, (2) individuals from underserved groups, and (3) their social networks. CBOs play an important public health function within underserved groups. They are well-entrenched within the communities they work with and thus would have greater credibility [49,50], a better grasp on the daily health and financial challenges faced by individuals in the communities, and an intuitive sense of what type of interventions would work. For individuals, it is critical that researchers start to think of the target audience of our eHealth interventions not as passive receivers of information and help but as active members of the communities they are in. Beyond reaching a specific individual, it is critical to engage people as a group, as research has shown that social capital and interpersonal communication can facilitate information sharing and trust and leverage collective actions to foster a sense of community when using eHealth interventions [27,29].

For PLANET MassCONECT, our strategy was to add value to CBOs by developing a web-based health portal that could function as a one-stop-shop by pulling public health data that would be relevant for their day-to-day work. However, we wanted to do so in a way that would organically foster a culture of learning that would be sustainable and continue even after our prescribed intervention period. To do so, we intentionally structured the web-based portal training to facilitate the fostering of social networks and strengthening of ties and relationships among staff members from different CBOs. Apart from the staff members receiving the most up-to-date knowledge and training in the use of web-based portals to enhance their work, the most valuable work was the creation of a network of dissemination specialists [39] where they could lean on each other for resources when the project ended.

For project SNAP, we attempted to leverage the ubiquity of mobile phones by promoting *citizen science* among our participants. Citizen science is a participatory approach where people are actively engaged in data collection and knowledge translation [51], as opposed to *subjects* where their role would be more passive. To understand the degree of disproportionate tobacco advertising targeted at underserved groups in Boston and Houston, we empowered our participants to take on the role of a collaborator in data collection, where they would snap photos of tobacco messages they encountered in their built environment for a period of 8 weeks. We also monitored their social interaction and how often they smoked together and engaged in conversations regarding smoking and quit attempts.

Although we advocate long-term collaborations with multiple stakeholders, we are mindful that the downsides of collaborations are that the formation of new relationships is resource intensive, and some stakeholders may ultimately not fulfill their obligations. To circumvent these problems, existing implementation science and public health research have advocated having the following three components to ensure that community engagement projects are sustainable over the long run: (1) institutionalized participation, (2) investment in communities, and (3) knowledge production and transfer.

In the context of eHealth intervention projects, although developing and managing relationships with stakeholders could be challenging, a structured and systematic approach to institutionalizing the participation of different stakeholders through formal agreements and engagement plans could provide a scaffold to support such collaborations. Moving beyond formal agreements, investing in communities that health organizations serve could foster greater trust among different stakeholders. These could be achieved by building capacity among different stakeholders, raising digital skills, creating collaborative networks and sharing resources, and empowering individuals. An example of this could be large anchor organizations (eg, large hospitals in communities) taking the lead and creating communities of practice, where other health organizations can come together periodically (eg, annually) to share best practices of using eHealth interventions to address health disparities and their challenges [17]. Finally, the knowledge gained from eHealth intervention projects should be translated into actual practice, and that is where smaller organizations with experience working with underserved groups could provide contextual insights on what works and under what circumstances.

## Principle 3: Design With Usability in Mind: Enhancing Readability and Navigability

One of the most difficult challenges in undertaking eHealth intervention design for underserved groups is that besides tackling issues pertaining to technology, design, or even costs, public health organizations, researchers, and technology developers need to understand how usable technologies are to underserved populations in their day-to-day lives. One of the pitfalls for public health researchers involved in building and leveraging communication technologies is having the implicit belief that having technology itself accompanied by the big data it collects as well as sophisticated artificial intelligence algorithms to analyze it would be the panacea in solving health problems brought about by disparities [52]. However, our experiences informed us that at the end of the day, usability is a major factor in technology adoption [53].

When we conducted usability testing for our C2C web portal, we found that one aspect of usability that was often overlooked was the *readability* of the information presented. We found that although individuals from underserved groups expressed interest in using health web portals, one of the main hindrances was site *readability*—whether the content is easily digestible by the intended participants. The participants had very low literacy skills and did not understand some of the languages presented on various pages of the site, although it was perceived as *simple* for the researchers. It became apparent that for the web portal intervention to work, the language would need to be simplified to a very basic level, and terms should reflect literal meanings (eg, no difficult words or metaphors). In addition, even when we provided links to external resources, these websites should be curated for the suitability of low literacy audiences. The focus on content and designing the portal, with readability as a priority, was a crucial factor determining its success through three pathways. First, it has been well established by research that when individuals pay attention to media content [30,54], they are more likely to gain knowledge. Second, research has shown that when individuals pay attention to content delivered via the internet (ie, web portals), they are more likely to engage with others in interpersonal discussion [30], eventually leading to the acquisition of health knowledge, as individuals within social networks help make sense and connect the dots. Third, websites may motivate elaborative processing, the act of connecting new pieces of information with one’s knowledge, associated with knowledge gains [31].

A second aspect of usability that researchers should pay attention to is the *navigability* aspect—the ease of traversing one page to another to look for information or activate certain functions. In our C2C web portal (Figure 2), we deliberately designed the main navigation panel to be situated on the left-hand side of the webpage without any top panels that one might see in other similar sites. In the original design, we had a top panel with links to our health content for participants and a left panel with links to external sites, similar to existing health portals. However, the participants were confused as to why there were 2 panels and often relied on the left panel bar, which only brought them to external sites, and they missed out on accessing the actual health content we had prepared for them, which was accessible only from the top panel. To achieve a parsimonious look and simplify use for our participants, we consolidated the navigation panel to the left side of the portal.

For the SNAP project, we found that it is critical to communicate successful navigation with clear visual cues when designing mobile apps for health, although it may be simple. One of the key features of the app was enabling participants to take photos of anti- or pro-tobacco messages they came across in their daily lives (eg, billboards, advertisements, and shops), which allowed us to map out hotspots of tobacco messaging [31]. In the earlier version of the app, participants needed to manually return to the home page of the app after submitting the photo. However, as they were not brought back to the main page directly, they were confused about whether their photo submissions were successful. Another key feature of our app was a snooze function that enabled participants to pause passive data collection by the app should they want privacy at any point in time. However, it was not clear to the participants if they were successful in pausing the data collection after hitting the snooze function, and some went to their phone settings to manually disable the geolocation tracking by the app. After the feedback, we designed a pop-out SMS text message that clearly highlighted that data collection was paused after they clicked the snooze function.

On the basis of our experiences, we list the following concrete steps that researchers could consider when implementing eHealth interventions in low-resource settings to enhance readability and navigability. First, we strongly recommend that usability testing sessions be designed to explicitly obtain data on how easy it is for participants from underserved groups to comprehend the health content administered through digital gadgets, as well as asking them to provide comments and feedback on how easy (or difficult) it is to navigate and use it. Second, in addition to structuring the usability sessions in a traditionally *passive* manner where participants simply perform the tasks required, researchers could adopt a *think-aloud* protocol of interviewing [55]. The think-aloud interview requires participants to engage in certain tasks: it could be reading content or navigating features of certain health technology and simultaneously expressing their thoughts on whether they found the tasks easy or difficult, which would be recorded [56]. Then, the data could be transcribed and analyzed quantitatively for all participants. In this way, gaps in readability and navigability could be systematically captured and analyzed, providing researchers with empirical insights into areas where significant changes should be made.

## Principle 4: Build Privacy Safeguards Into eHealth Interventions and Communicate Privacy-Utility Tradeoffs in Simplicity

With the increasing amount of data collected through eHealth interventions and the public scandals around privacy intrusion and data breaches, it is critical that researchers make privacy and ethics a conversation to have with participants right from the get-go. As the smartphone app used in our SNAP project aimed to collect high-density spatiotemporal data from participants ranging from their web searches to geolocations and the social networks they came into contact with, which are highly personal, we aimed to communicate privacy protection measures to our participants even at the app design stage.

There were several levels of privacy safeguards that were put into place. On a macro level, the app was compliant with the General Data Protection Regulation requirements [52,57], where participants had the right to access or delete their data. In addition, the app was programmed to collect data passively as participants went about their daily lives with a snooze function that they could turn on if they wanted to stop data collection temporarily for any reason.

Although these safeguards were in place, we found that the biggest gap was in the area of communication of privacy measures. During our usability testing, many participants did not understand the function of the snooze button and why it was necessary; many, in fact, suggested removing the snooze function altogether. This highlighted a difference in how participants and researchers approached issues of privacy and indicated to us that some participants might not fully grasp concepts of data privacy, protection, and rights and ownership that we as eHealth interventions researchers are so used to. As such, we recommend that researchers intentionally involve participants from underserved groups in designing privacy features through either focus group or usability testing sessions. This would allow researchers to hear the participants’ concerns. In other words, researchers should dedicate a portion of the eHealth intervention training to explain clearly what privacy is, why it matters to the participants, what data are collected, what are the rights over their data, and how they could exercise those rights.

## Principle 5: Strive for an Optimal Balance Between Open Science Aspirations and Protection of Underserved Groups

Finally, in the field of eHealth interventions, it is inevitable for researchers to engage in the conversation between the need to strive for *open science* and protect the voluminous amount of data obtained from communication technologies used in the interventions. The push for researchers to adopt the open science movement is a fairly recent phenomenon, driven in part by the rise in big data and artificial intelligence research [58]. Researchers who subscribe to the open science movement aim to foster collaborative networks across research institutions and countries by promoting a culture of *openness* in sharing data and algorithms alongside publications. There are many advantages to the open science paradigm, which address the replicability crisis in social science by enabling other researchers to reproduce similar results with the same data and code, prevent questionable research practices such as p-hacking, and promote best practices of code writing and analysis where others could see and provide constructive criticisms on best practices for data preparation as well as code writing [59].

For many researchers who work in the intersection between eHealth interventions and health promotion among underserved populations, such as ours, we find ourselves in an ideological conundrum. As social scientists, we aspire toward the rigor and collaborative culture of the open science paradigm; on the other hand, we are bound by our commitment to protectively guarding the data collected from underserved groups, knowing that the data could further penalize them if they fall into the wrong hands. For instance, we could track the websites that our C2C participants visited, although they may not be directly related to the project. Through our SNAP project, we collected data at a granular level where we could know and map individuals’ web-browsing patterns, know where they travel to (down to a specific latitude and longitude), and gain insights into the frequency of social contact with their friends captured by Bluetooth data, which provided information when our participants were physically co-located in a given environment. If malicious actors or organizations gain access to these data, they could capitalize on the insights drawn from the data at the expense of people from lower socioeconomic groups.

Although we as a community are still learning to balance this tension, there are several practical steps that one could take to achieve a reasonable balance between embracing open science and data protection. The most basic step researchers could take is to always aggregate data so that sensitive details of individuals would retain a higher level of anonymity. For our C2C intervention, although we could potentially track the web-browsing behaviors of underserved populations, we chose to collect data at the household (ie, aggregate) level, knowing that this tradeoff would provide an additional layer of protection for our participants. As for geolocations captured through smartphones in our SNAP study, although we could analyze the data at the most granular level (eg, the exact locations they have visited down to the seconds), we collapsed the geolocations into 10-minute bins for analyses, making it potentially more difficult to trace the geolocation patterns to specific individuals. Analyzing data at the aggregated level gave us the confidence to present sensitive information (eg, hotspot maps) without compromising the privacy of our participants.

A second practical way to balance the tension between open science and participants’ privacy is to host the data and analyze them through a secure remote data storage and analysis environment as much as possible, as opposed to storing and analyzing data in researchers’ desktops in the office or personal laptops. This is part of good data hygiene; after all, the security of personal desktops and laptops has a higher likelihood of being compromised compared with a secure cluster computing environment. For instance, we relied on the remote computing environment provided by the Institute of Quantitative Social Sciences at Harvard University for data management and analysis of part of our SNAP data. Apart from the universities’ cluster computing environment, researchers may seek out trustworthy companies involved in cloud computing to curate their data management and analysis pipeline.

Third, researchers could promote or engage in *selective openness* depending on the nature of their eHealth interventions. For our capacity-building work with CBOs in Planet MassCONECT, the sharing of data and best practices were done in a safe zone, where the audiences are staff members from different CBOs involved in similar work with underserved groups. Thus, openness is achieved in an environment of trust and familiarity, where information sharing on best practices would benefit all CBOs. At the same time, by only sharing data and information with those invested in the communities, it prevents external organizations or individuals from accessing sensitive data.

## Limitations

We are mindful that while we aim to provide a synthesis of the principles over the decade, there are several limitations. First, we recognize that every research team and organization working with underserved groups have unique challenges and that some of our recommendations may not be directly applicable. As such, it is important for research teams to be keenly aware of their own unique contexts and only apply those strategies and principles that are most relevant. Second, as most of our work was conducted in the United States, there may be cultural barriers specific to different countries. For instance, cultural worldviews such as fatalism may further impede eHealth intervention adoption because of the inherent belief that there was nothing that patients could do to avoid contracting certain diseases [29,60].

## How the Proposed Principles Complement Existing Knowledge in eHealth Interventions

Despite the limitations, there are several ways in which the proposed principles will address gaps in knowledge within existing eHealth intervention research. Currently, research on eHealth interventions in the context of health disparities typically focuses on improving usability design [61], identifying factors to improve adoption [3], and how digital health technologies may amplify inequality [6]. We build upon the current knowledge of eHealth interventions in a few ways. First, we argue that the success of eHealth interventions goes beyond usability and interface issues, and researchers and technology developers need to pay attention to the broader context that would influence the eventual adoption and use of digital health technologies. Second, we strongly advocate that eHealth interventions will need to be embraced and supported by multiple stakeholders over the long run to have a tangible impact on the health of underserved groups. This would involve building networks and technological capacity among under-resourced CBOs so that they could effectively leverage eHealth interventions in their daily work. Finally, we highlight the need for the research community to be transparent and address privacy concern issues collectively with participants from the get-go to balance the benefits of open science and protect the data of communities that embrace the use of these technologies. Future research could extend our research by studying the broader contextual factors that facilitate or impede the use of eHealth interventions by health researchers using a case study approach [62]. This would enable researchers to gain a holistic understanding of the specific opportunities and constraints faced by organizations in eHealth intervention research. In addition, future scholars could conduct longitudinal field experiments to ascertain the effectiveness of eHealth interventions in addressing health disparities over the long run, as well as to systematically examine how openness and transparency with privacy issues may improve trust and adoption of eHealth interventions among underserved groups.

## Conclusions

There has never been a more exciting time to be involved in the development and implementation of eHealth interventions among underserved groups. With advancements in big data platforms and artificial intelligence, there are multiple opportunities to leverage technologies and data to improve health for the underserved [17]. However, the aspects of technology design and data analysis are small puzzles in the scheme of the big picture. The implementation of successful eHealth interventions ultimately rests on how well researchers understand the barriers to and facilitators of technology acceptance. Beyond that, researchers need to understand the digital media landscape of underserved populations, navigate and grasp sociocultural norms, as well as bring organizations, communities, and individuals on the same page to address health disparities through technology.

## Appendix

Multimedia Appendix 1 Summary of eHealth intervention projects in the Viswanath laboratory.

## Abbreviations

C2C: Click to Connect
CBO: community-based organization
EHR: electronic health record
SEP: socioeconomic position
SNAP: Smartphone App for Public Health
