# Supplementary material for: Designing Effective eHealth Interventions for Underserved Groups: Five Lessons From a Decade of eHealth Intervention Design and Deployment
Source: J Med Internet Res. 2022 Jan 7;24(1):e25419. doi: 10.2196/25419 (PMC8783288; doi:10.2196/25419)
Supplement: Multimedia Appendix 1 [file jmir_v24i1e25419_app1.docx]

**Table 1.** Summary of eHealth intervention projects in the Viswanath laboratory.

| Project and objectives | | Target group | Methods | Lessons learned | Principles |
| --- | --- | --- | --- | --- | --- |
| **Click to connect (C2C)** | | | | | |
|  | Improve eHealth literacy among people from low socioeconomic position (SEP) groups | Low SEP individuals recruited from adult education centers in General Educational Development, pre-General Educational Development, or English for speakers of another language classes | - Develop a web health portal - Purchase and provide computer and broadband internet access for the entire length of study - Conduct training classes where participants were taught digital skills such as how to use computers and the internet - Ongoing technical support if participants had any questions on the health webportal or connectivity issues | - Arrange for face-to-face contact with community leaders and organizations, as well as prepare recruitment presentations and meetings in the community - Provide the health technologies required for interventions (eg, computers, webportals, and smartphone apps) as well as defray the costs of using them (eg, broadband internet access) - Ensure that content on websites built for underserved groups is easily understood - Collect data at the aggregate rather than individual level | - Develop a strategic road map to address communication inequalities - Engage multiple stakeholders from the beginning for the long haul - Design with usability—readability and navigability—in mind - Build privacy safeguards into eHealth interventions and communicate privacy–utility tradeoffs in simplicity, as well as striving for an optimal balance between open science aspirations and protection of underserved communities |

| **PLANET MassCONECT** | | | | |
| --- | --- | --- | --- | --- |
| Build capacity for systematic program planning among a diverse range of CBOs^a^ working with the underserved population in Massachusetts | CBOs that conduct health outreach in Boston, Lawrence, and Worcester in the state of Massachusetts | - Develop a web-based health portal containing health resources (www.planetmassconnect.org) - Implement a 2-day capacity-building workshop for CBOs - Provide training manuals, handouts, and case studies - Highlight potential pilot grants to apply newfound knowledge - Facilitate networking opportunities to promote learning networks in which trainees can support each other | - Provide the health technologies required for interventions (eg, computers, webportals, and smartphone apps), as well as defray the costs of using them (eg, broadband internet access) - Empower CBOs by developing a web-based health portal that could function as a one-stop-shop by compiling public health data relevant to their day-to-day work - Create networks among CBOs so that they can leverage each other’s strengths to reach underserved groups - Encourage sharing of data and best practices were done in a safe zone, where the audiences are staff members from different CBOs involved in similar work with underserved communities | - Develop a strategic road map to address communication inequalities - Design with usability—readability and navigability—in mind - Engage multiple stakeholders from the beginning for the long haul - Strive for an optimal balance between open science aspirations and protection of underserved communities |

| **SNAP^b^** | | | | | |
| --- | --- | --- | --- | --- | --- |
|  | Leverage smartphone capabilities in collecting data passively and actively to measure exposure to tobacco messages in a built environment | Youths (aged 18-34 years) from underserved communities in the cities of Boston, Lawrence, and Houston | Ecological momentary assessment using a mobile app called Ethica | - Recruit individuals and their social networks to take part in the study together - Provide the health technologies required for interventions (eg, computers, webportals, and smartphone apps) as well as defray the costs of using them (eg, broadband internet access) - Allow participants to co-design features in the mobile health app - Bring privacy issues to the forefront and allow participants to articulate their concerns | - Engage multiple stakeholders from the beginning for the long haul - Develop a strategic road map in addressing communication inequalities - Design with usability—readability and navigability—in mind - Build privacy safeguards into eHealth interventions and communicate privacy–utility tradeoffs in simplicity |

^a^CBO: community-based organization.

^b^SNAP: Smartphone App for Public Health.
